# Computed Tomography (CT) Imaging Features of Patients with COVID-19: Systematic Review and Meta-Analysis

**DOI:** 10.1155/2020/1023506

**Published:** 2020-07-23

**Authors:** Ephrem Awulachew, Kuma Diriba, Asrat Anja, Eyob Getu, Firehiwot Belayneh

**Affiliations:** Dilla University, College of Health Science and Medicine, Dila, Ethiopia

## Abstract

**Introduction:**

Severe acute respiratory syndrome coronavirus 2 (SARS-CoV-2) is a highly contagious disease, and its first outbreak was reported in Wuhan, China. A coronavirus disease (COVID-19) causes severe respiratory distress (ARDS). Due to the primary involvement of the respiratory system, chest CT is strongly recommended in suspected COVID-19 cases, for both initial evaluation and follow-up.

**Objective:**

The aim of this review was to systematically analyze the existing literature on CT imaging features of patients with COVID-19 pneumonia.

**Methods:**

A systematic search was conducted on PubMed, Embase, Cochrane Library, Open Access Journals (OAJ), and Google Scholar databases until April 15, 2020. All articles with a report of CT findings in COVID-19 patients published in English from the onset of COVID-19 outbreak to April 20, 2020, were included in the study.

**Result:**

From a total of 5041 COVID-19-infected patients, about 98% (4940/5041) had abnormalities in chest CT, while about 2% have normal chest CT findings. Among COVID-19 patients with abnormal chest CT findings, 80% (3952/4940) had bilateral lung involvement. Ground-glass opacity (GGO) and mixed GGO with consolidation were observed in 2482 (65%) and 768 (18%) patients, respectively. Consolidations were detected in 1259 (22%) patients with COVID-19 pneumonia. CT images also showed interlobular septal thickening in about 691 (27%) patients.

**Conclusion:**

Frequent involvement of bilateral lung infections, ground-glass opacities, consolidation, crazy paving pattern, air bronchogram signs, and intralobular septal thickening were common CT imaging features of patients with COVID-19 pneumonia.

## 1. Introduction

Severe acute respiratory syndrome coronavirus 2 (SARS-CoV-2) is a highly contagious disease, and its first outbreak was reported in Wuhan, China [1]. On January 30, 2020, the World Health Organization (WHO) declared it a pandemic disease [2]. Currently, the disease has been reported in more than 212 countries worldwide [3]. As of May 01, a total of 3,325,620 cases and 234,496 deaths due to COVID-19 were reported worldwide [4]. The most common diagnostic tool for coronavirus disease 2019 (COVID-19) infection is real-time polymerase chain reaction (RT-PCR), which is regarded as the reference standard [5, 6].

COVID-19 causes severe respiratory distress (ARDS). Due to the primary involvement of the respiratory system, chest computed tomography (CT) is strongly recommended in suspected COVID-19 cases, for both initial evaluation and follow-up [7]. Recent studies addressed the importance of chest CT examination in COVID-19 patients with false-negative RT-PCR results [8] and reported the CT sensitivity as 98% [9]. Additionally, CT examinations also have great significance in monitoring disease progression and evaluating therapeutic efficacy.

The SARS-CoV-2 has four major structural proteins: the spike surface glycoprotein, small envelope protein, matrix protein, and nucleocapsid protein [10]. The spike protein binds to host receptors via the receptor-binding domains (RBDs) of angiotensin-converting enzyme-2 (ACE2) [11]. The ACE2 protein has been identified in various human organs, including the respiratory system, GI tract, lymph nodes, thymus, bone marrow, spleen, liver, kidney, and brain. SARS-CoV-2 was reported to utilize angiotensin-converting enzyme-2 (ACE2) as the cell receptor in humans [12], firstly causing pulmonary interstitial damage and subsequently with parenchymal changes. Reportedly, chest CT images could manifest different imaging features or patterns in COVID-19 patients with different time course and disease severity [13, 14].

Studies suggest that routine chest CT is a useful tool in the early diagnosis of COVID-19 infection, especially in settings of limited availability of reverse-transcriptase polymerase chain reaction (RT-PCR [15]. Imaging is critical in assessing severity and disease progression in COVID-19 infection. Radiologists should be aware of the features and patterns of imaging manifestations of the novel COVID-19 infection. A variety of imaging features have been described in similar coronavirus-associated syndromes. Due to an alarming spread of COVID-19 outbreak throughout the world, a comprehensive understanding of the importance of evaluating chest CT imaging findings is essential for effective patient management and treatment. Individual literature published is required to be summarized. Thus, a comprehensive systematic review has to be performed. Therefore, this study systematically reviewed CT imaging features of patients with COVID-19 pneumonia.

## 2. Methods

### 2.1. Search Strategy

A systematic search was conducted on PubMed, Embase, Cochrane Library, Open Access Journals (OAJ), and Google Scholar databases until April 15, 2020. Keywords used to search eligible studies were “severe acute respiratory syndrome 2,” “SARS CoV 2,” “2019-nCoV,” “COVID-19,” “computed tomography,” “CT,” and “radiology.” All identified keywords and mesh terms were combined using the “OR” operator and “AND” operator for searching literatures. We simultaneously searched the reference lists of all recovered articles for potentially eligible studies.

### 2.2. Eligibility Criteria

All articles with a report of CT findings in COVID-19 patients published in English from the onset of COVID-19 outbreak to April 20, 2020, were included in the study. Case studies and case series reported on chest CT imaging findings of patients with COVID-19 were included with caution. Studies pertaining to other coronavirus-related illnesses were excluded.

### 2.3. Assessment of Study Quality

Studies selected for inclusion were assessed for methodological quality by two teams of independent reviewers using the standard critical appraisal instruments of the Joanna Briggs Institute Meta-Analysis of Statistics Assessment for the Review Instrument (JBI-MAStARI) [16]. Disagreements were resolved by consensus.

### 2.4. Data Extraction and Synthesis

Data were extracted by two teams of the investigators using a standardized data extraction form. In addition, eligible case studies and case series studies were extracted carefully. Then the extracted data were merged for systematic analysis. The main outcomes extracted from each study were study design, country, patient demographics, and chest CT findings. Additional findings extracted were the patient's clinical characteristics. Disagreements were discussed with other reviewers and subsequently resolved via consensus.

### 2.5. Data Analysis and Data Synthesis

Systematic reviews and meta-analyses were carried out using R software version 3.6.1 with user-contributed commands for meta-analyses: metaprop, metan, metainf, metabias, and metareg. The effect sizes and SEs of the studies were pooled using a random-effects model to calculate the pooled estimates of CT findings among COVID-19 patients. A meta-analysis was also planned to assess the association of various imaging findings with demographic data.

### 2.6. Risk of Bias and Sensitivity Analysis

Evidence for statistical heterogeneity of the results was assessed using the Cochrane Q *x*2 test and *I*2 statistic. A significance level of *P* < 0.10 and *I*2 >50% was interpreted as evidence of heterogeneity [17]. A potential source of heterogeneity was investigated by subgroup analysis and meta-regression analysis [18]. Where statistical pooling was not possible, the findings were presented in a narrative form including tables and figures to aid in data presentation where appropriate.

Sensitivity analyses were conducted to weigh up the relative influence of each individual study on the pooled effect size using a user-written function, metainf. The presence of publication bias was assessed informally by visual inspection of funnel plots [19].

## 3. Result

### 3.1. Study Selection

**Figure 1:**
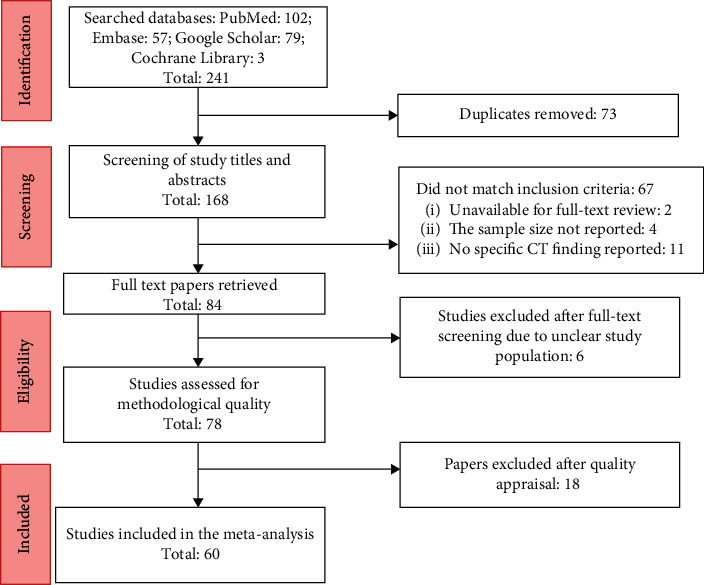
Flow chart of the search and study inclusion.

Following the initial search, 241 studies were identified from the electronic databases (Figure 1). After the removal of 67 duplicates, 17 noneligible studies, and six studies with unclear population, 78 studies were retrieved for full-text review. Of this, 18 studies were of poor quality that did not meet the eligibility criteria. Following methodological quality assessment, 60 articles were included in the meta-analysis. In the included studies, a total of 5041 patients with COVID-19 were assessed for CT imaging features. All papers were published in English.

### 3.2. Study Characteristics

In this review, 60 papers were eligible and a total of 5041 patients had CT imaging findings [8, 12–14, 19–72]. From a total of 5401 participants, about half of them were male 2710 (50.2%), while 2693 (49.8%) were female. The mean age of the participants was 49 years with a standard deviation of 11.6 years.

#### 3.2.1. Clinical Features of Patients with COVID-19

From a total of 60 included studies, only 48 studies had reported the clinical features of COVID-19 infected patients. According to the report of 48 studies, the main clinical features of COVID-19 infected patients were fever and dry cough, which accounted for 80% (2954/3800) and 56.2% (2137/3800), respectively, while about 2.3% (86/3800) patients with COVID-19 infection are asymptomatic. The total white blood cell count was decreased in about 24% (410) of patients with COVID-19, while about 2% [43] had increased white blood cells. Decreased lymphocyte count was detected in about 442 (43%) patients with COVID-19, and only 1% had increased lymphocyte count. Markers like C-reactive protein were found to be increased in about 87% (458) patients, while only 13% (254) had normal C-reactive protein (Table 1).

#### 3.2.2. Chest CT Imaging Features of COVID-19-Infected Patients

**Figure 2:**
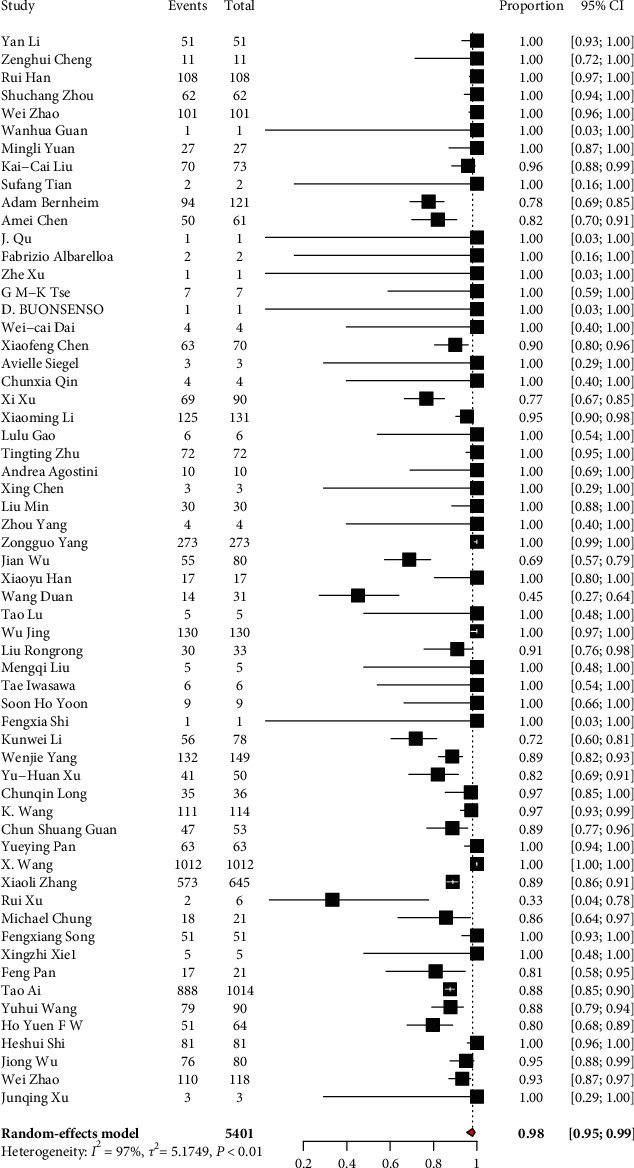
Magnitude of abnormal chest CT finding of patients with COVID-19.

We performed meta-analyses of primary outcome data and secondary outcomes with available data. From a total of 5041 COVID-19-infected patients, about 98% (4940/5041) had abnormalities in chest CT, while about 2% had normal chest CT findings (Figure 2). Among COVID-19 patients with abnormal chest CT findings, 80% (3952/4940) had bilateral lung involvement. About 20% (641/3206) patients had exclusively unilateral lung involvement. When a single lobe was involved, the right lung was most often affected (62%) and 38% of them had only left lung involvement. In the right lung, the lower lobe was frequently involved (74% (784)), while the middle lobe was less frequent (38% (326)). In the left lung, the upper lobe was frequently involved (74% (731)).

Regarding the patterns of lung lesions in patients with COVID-19, ground-glass opacity (GGO) and mixed GGO with consolidations were observed in 2482 (65%) and 768 (18%) patients, respectively. Consolidation, which is defined as denser opacities and blurred margins of pulmonary blood vessels and bronchial tubes, was detected in 1259 (22%) patients with COVID-19 pneumonia. CT images also showed interlobular septal thickening in about 691 (27%) patients. CT showed that 11 (21.6%) patients had discrete pulmonary nodules. Crazy paving patterns of lesions were observed in 575 (12%) patients. Five hundred thirty-one (18%) patients had an air bronchogram sign. Pleural effusion and lymphadenopathy were observed in 94 (1.6%) and 21 (0.7%) patients with COVID-19 pneumonia, respectively. Pulmonary nodules were reported in 262 (9%) patients (Table 2).

### 3.3. Additional Analysis

We also gathered data to demonstrate if there is a difference in the CT findings related to infection time course. In the included studies, the mean time between initial chest CT and follow-up was about 6.5 days (range, 0–21 days). According to the report of 9 included studies, 43% (95 CI: 26%–61%) patients have improved follow-up chest CT findings, while 30% (95 CI: 18%–46%) have advanced chest CT findings [40, 43, 44, 50, 52, 57, 64–66]. Our findings demonstrated pure ground-glass opacity in early disease, followed by the development of crazy paving, and finally increasing consolidation latter in the disease course. GGO density increased and transformed into consolidation; consolidation edges were flat or contracted; and fibrous cord shadow appeared. GGO with consolidation was higher in the advanced stage than in the early stage of the disease (OR 3.2, 95% CI: 2.2–4.7, *P*=0.013). A crazy paving pattern and a reverse halo sign were all rare in the early sign, but were present in the late stage of the disease. In terms of distribution of disease, bilateral involvements were prominent in the later stage than in the early stage (*P* < 0.001). According to the report of two included studies, pure GGO was the common imaging feature of mild and moderate stages of the disease course, while consolidation and GGO with consolidation increased by 64% and 79% in the critical stage of the disease, respectively [57, 58].

### 3.4. Heterogeneity and Risk of Bias

Subgroup analysis was conducted to justify the cause of heterogeneity. Subgroup analysis of the included studies showed that the possible cause of heterogeneity was sample size (*P* value < 0.01). Funnel plots did not suggest a publication bias for the majority of the parameters. We demonstrated no publication bias (*P* value = 0.1947).

## 4. Discussion

### 4.1. Summary of Evidence

In this review, we have demonstrated that the main signs and symptoms of hospital admission of patients with COVID-19 pneumonia were fever, dry cough, fatigue, pharyngeal pain, and respiratory distress. About 24% of the patients had reduced total leukocyte count and 43% had reduced lymphocyte count. Although the laboratory findings are not specific for viral pneumonia, leukopenia and lymphocytopenia may be helpful to distinguish COVID-19 from common bacterial infections. The majority of the patients had increased C-reactive protein.

In this study, common CT imaging features in patients with COVID-19 pneumonia included bilateral involvement, ground-glass opacities, consolidation, crazy paving pattern, air bronchogram signs, and intralobular septal thickening. At a later stage of the disease, mixed GGO with consolidation was the more frequent finding. Pulmonary consolidation was mainly found in the severe and progressive late stages of the disease, which can coexist with ground-glass and fibrotic changes. The pathological basis of these changes could be due to inflammatory cell infiltration and interstitial thickening, cell exudation, and hyaline membrane formation.

In the present study, the increased frequency of GGO, consolidation, bilateral disease, intralobular septal thickening, a crazy paving pattern of the lesion, and appearance of reverse halo sign lesions over more than one lobe of the lung could represent the pathophysiology of the disease process as it organizes and it could also probably explain chest CT hallmark of COVID-19 infection. CT imaging findings can be correlated with the severity of the disease and disease progression after efforts of treatment.

The majority of the patients (76%) had multilobar involvement, and lesions were more frequent in the right lung. Pleural effusion, lymphadenopathy, and pulmonary nodules were less common imaging findings in these patients. About 2% of the patients had normal initial CT findings. When a single lobe was involved, the right lung was most often affected and more than half of the patients with COVID-19 had multiple lobe infections. In the right lung, the lower lobe is frequently involved while the middle lobe was less frequent. In the left lung, the upper lobe was frequently involved. This might indicate the virus tends to disseminate all over all lobes of both lungs as the disease progress. Chest CT imaging features may avoid repeated laboratory testing and may be helpful in a resource-limited country.

According to the present study, chest CT imaging showed similar characteristics in the majority of patients, including predominantly bilateral and multilobe involvement. The pattern of ground-glass and consolidative pulmonary opacities, often with a bilateral lung distribution, is somewhat similar to that described in earlier coronavirus outbreaks such as SARS and MERS, which was known to cause ground-glass opacities that may coalesce into dense consolidative lesions [73, 74].

According to the report of 9 included studies, 43% (95 CI: 26%–61%) patients have improved follow-up chest CT findings, while 30% (95 CI: 18%–46%) have advanced chest CT findings. Our findings demonstrated pure ground-glass opacity in early disease, followed by the development of crazy paving and finally increasing consolidation latter in the disease course. According to the report of two included studies, pure GGO was the common imaging feature of mild and moderate stages of the disease course, while consolidation and GGO with consolidation increased by 64% and 79% in the critical stage of the disease, respectively.

### 4.2. Limitation

This systematic review and meta-analysis came up with CT imaging features of patients with COVID-19; we acknowledge a few limitations of the present systematic review and meta-analysis, which may affect the results. First of all, two relevant studies which were identified through our literature search were excluded due to unavailability for full-text review. The other limitation of the present study was that it was limited to articles published in English.

## 5. Conclusions

The present study showed that common CT imaging features of COVID-19 pneumonia included frequent involvement of bilateral lung infections, ground-glass opacities, consolidation, crazy paving pattern, air bronchogram signs, and intralobular septal thickening. Bilateral involvement was common while single lobe involvement was rare. This sign of CT imaging might be an important tool for diagnosis and monitoring disease progression in patients with COVID-19 infection.

## Figures and Tables

**Table 1:** Review of clinical features of patients with COVID-19.

Clinical features	Number of studies	Number of patients (%)
Sign and symptoms		
Fever	48	2954 (80%)
Dry cough	48	2137 (56.2%)
Respiratory distress	29	462 (15%)
Pharyngeal pain	48	381 (13%)
Fatigue	32	481 (27%)
Total WBC count		
Normal	22	1195 (68%)
Decreased	22	410 (24%)
Increased	22	45 (2%)
Lymphocyte count		
Normal	22	566 (56%)
Decreased	22	442 (43%)
Increased	22	2 (1%)
C-reactive protein		
Normal	15	254 (13%)
Decreased	15	0 (0%)
Increased	15	458 (87%)

**Table 2:** CT imaging features of patients with COVID-19.

CT findings	Number of studies	Number of patients (%)
Patterns of the lesion		
Ground-glass opacity with consolidation	60	768 (18%)
Ground-glass opacity	60	2482 (65%)
Consolidation	60	1259 (22%)
Crazy paving pattern	24	575 (12%)
Reversed halo sign	24	146 (1%)
Other signs in the lesion		
Interlobular septal thickening	23	691 (27%)
Air bronchogram sign	23	531 (18%)
Distribution		
Bilateral	48	3952 (80%)
Unilateral	48	641 (20%)
Right lung	8	48 (62%)
Left lung	8	29 (38%)
Number of lobes involved		
One lobe	13	278 (14%)
Two lobes	13	299 (11%)
Three lobes	13	250 (13%)
Four lobes	13	212 (15%)
Five lobes	14	384 (34%)
More than one lobe	14	1145 (76%)
Lobe of lesion distribution		
Left upper lobe	14	731 (74%)
Left lower lobe	20	504 (46%)
Right upper lobe	19	455 (40%)
Right middle lobe	15	326 (38%)
Right lower lobe	17	784 (74%)
Other findings		
Pleural effusion	60	94 (1.6%)
Lymphadenopathy	60	21 (0.7%)
Pulmonary nodules	22	262 (9%)

## Abbreviations

ACE2: Angiotensin-converting enzyme-2
ARDS: Acute respiratory distress
CT: Computed tomography imaging
COVID-19: Coronavirus disease 2019
GGO: Ground-glass opacity
2019-nCoV: Novel coronavirus
SAR CoV 2: Severe acute respiratory syndrome coronavirus 2.
